# Baseline knowledge, attitudes and perceptions on the oncology profession among final-year medical students in Kenya: a cross-sectional study

**DOI:** 10.3332/ecancer.2026.2146

**Published:** 2026-06-11

**Authors:** Mustafa Affey, Najma Mahamed Aden, Jamal Saman, Fatuma Affey, Abdirahman Maalim, Ibrahimrashid Mohamed Sheikh, Mohamedamin Abdullahi, Omar Abdihamid

**Affiliations:** 1 Faculty of Health Sciences, Kenyatta National Hospital, PO BOX 19676-00202, Nairobi, Kenya; 2 School of Health Sciences, Kenyatta University, PO BOX 43844-00100, Nairobi, Kenya; 3 Institute of Research and Development, Benadir University, Mogadishu, Somalia; 4 School of Pure and Applied Sciences, Garissa University, PO Box 1801-70100, Garissa, Kenya; 5 Department of Medicine, School of Medicine, Moi University, PO Box 4606 - 30100, Eldoret, Kenya; 6 Department of Orthopaedic Surgery, Chelsea and Westminster Hospital, 369 Fulham Road, SW10 9NH London, UK; 7 Royal Bournemouth Hospital, NHS Foundation Trust, Dorset, UK; 8 Garissa Regional Cancer Center, Garissa County Referral Hospital, PO Box 29, Garissa, Kenya

**Keywords:** medical students, oncology, career, Kenya

## Abstract

**Background:**

Kenya faces a rising cancer burden amid a critical shortage of oncology professionals. Undergraduate exposure, knowledge and attitudes shape specialty choices, yet local evidence remains scarce. This study assessed baseline knowledge, attitudes and perceptions of oncology among final-year medical students in Kenya to inform curriculum development, mentorship and workforce planning.

**Methods:**

A descriptive cross-sectional online survey was conducted among final-year Bachelor of Medicine and Bachelor of Surgery students across accredited Kenyan medical schools between April and May 2025. A structured Qualtrics questionnaire assessed sociodemographics, career intentions, oncology knowledge and exposure and attitudes toward oncology. Data were analysed using descriptive statistics. Participation was voluntary and anonymous. Ethical approval was obtained from the Mount Kenya University Ethics Review Committee (MKU/ISERC/5342).

**Results:**

Of 89 responses, 88 were analysed. Participants were equally male and female (50% each), with a mean age of 25 years. Most respondents were from the University of Nairobi (73%), followed by Moi University (17%) and other institutions. Seventy-seven percent reported a close friend or relative diagnosed with cancer. Community responses were mainly supportive (60%), though 32% viewed cancer as a ‘death sentence’ and 8% reported stigma or avoidance. Personal or family experience shaped perceptions for 61% and 81% cited cultural beliefs as influencing community reactions. Seventy-three percent had completed an oncology rotation, but only 30% attended oncology-focused workshops. Over half (53%) were unsure of cancer incidence in Kenya. Chemotherapy and radiotherapy were the most recognised treatments, with limited awareness of immunotherapy. Eighty-six percent agreed that oncology training should be strengthened. While 84% knew oncology is a recognised specialty in Kenya, only 42% were aware of local postgraduate programs. Most rated the undergraduate oncology curriculum as ‘average’ (42%). Twenty-five percent would consider oncology as a career, 51% were undecided and 24% would not. Motivators included clinical exposure and patient outcomes; deterrents were emotional burden, lack of mentorship, patient suffering and poor work–life balance. Suggested strategies included increased clinical exposure, structured mentorship, expanded residency slots and targeted incentives.

**Conclusion:**

Final-year Kenyan medical students acknowledge oncology as a career but face significant knowledge and exposure gaps. Strengthening undergraduate oncology education, expanding mentorship opportunities and creating clear postgraduate pathways are key to building a robust, homegrown oncology workforce in Kenya.

## Background

The GLOBOCAN 2022 data estimates on cancer incidence, mortality and prevalence across 185 countries and 36 major cancer types show that breast cancer continues to be the most frequently diagnosed cancer among women worldwide (about 2.3 million new cases in 2022), followed by lung and cervical cancers, while lung and prostate cancers remain the predominant diagnoses among men [1]. About one in five people will develop cancer in their lifetime, highlighting the scale of the problem and the demand for cancer services and the need for more oncology workforce to meet this demand [2]. In Kenya, according to the most recent GLOBOCAN 2022 report, approximately 44,700 new cancer cases and 29,300 deaths due to cancer across all cancer types, with the leading cancers in Kenya being breast, cervical, prostate and esophageal cancers [3].

However, this global cancer burden disproportionately affects low- and middle-income countries (LMICs), where mortality rates remain high due to inadequate healthcare resources and workforce constraints [4]. A major barrier to effective cancer control in these countries is the shortage of a well-trained oncology workforce, making oncology education and training initiatives a critical component in building this capacity [5]. A scoping review on oncology training initiatives shows that Kenya, like many LMICs, faces critical shortages in oncology specialists (medical, surgical, radiation oncologists, pathologists and oncology nurses. Local initiatives, often in partnership with institutions from high-income countries, are attempting to bridge this gap by providing targeted graduate training and e-learning modules. However, sustainability remains a challenge and most programs are still short-term and externally driven [5].

Another global study on the cancer care workforce in Africa reveals that many countries have only a handful of oncologists, and some have none at all. Therefore, oncologists practicing in Africa face significantly heavier clinical workloads than their global counterparts, with a median of 325 new patient consults per year compared to 175 elsewhere, and nearly one-third seeing over 500 new patients annually. This overwhelming demand is compounded by limited infrastructure, restricted access to chemotherapy and radiotherapy, and lower job satisfaction, which collectively threaten the sustainability of cancer care systems on the continent [6]. In Kenya, these challenges are reflected in the limited oncology workforce, with fewer than 100 practicing oncologists serving a population of over 50 million people, highlighting the urgent need for expansion of local training programs and investment in retention strategies to meet the growing cancer burden [7, 8].

Worldwide, final-year medical students gravitate toward the traditional core specialties such as internal medicine, surgery, paediatrics and obstetrics/gynaecology, often guided by perceived prestige, lifestyle or mentorship, with limited interest in fields like oncology [9]. In Africa, such patterns are similarly prevalent due to a lack of structured oncology exposure, contributing to poor representation of oncology in students’ career choices as reported by a study from Nigeria [10]. A mixed-methods study at Muhimbili University of Health and Allied Sciences in Tanzania found that although 86% of medical students and 78% of nursing students had minimal cancer training, the vast majority (92%) expressed a positive attitude toward integrating oncology into the undergraduate curriculum, indicating strong interest in learning about the specialty [11].

In Kenya, medical students show limited interest in less traditional and emerging fields such as oncology, with most final-year students gravitating toward core specialties like internal medicine, surgery, paediatrics and obstetrics/gynaecology, which together accounted for nearly 60% of career preferences in one study [12]. This concentration of career choices highlights the challenge of building a sustainable oncology workforce, as few students pursue specialties aligned with cancer care, despite the country’s growing cancer burden and acute shortage of oncologists.

Kenya is addressing its oncology workforce crisis through the 2019–2030 National Cancer Action Plan, which prioritises training and regional cancer centers such as Garissa to reduce geographic disparities. University partnerships, including the Merck–Nairobi–Tata Fellowship, aim to expand the pool of specialists, while facilities like Kenyatta National Hospital now provide integrated oncology services closer to trainees. Despite progress, oncology remains underrepresented among Kenyan medical graduates. Understanding students’ baseline knowledge, attitudes and intentions is critical, as early perceptions shape career choices and can be influenced by mentorship, structured rotations and interest groups.

Therefore, this study assesses the perception, attitudes and baseline knowledge of oncology among final-year medical students to guide curriculum reforms, mentorship and policy, aligning workforce development with national cancer control priorities.

## Methods

### Study design and setting

We conducted an exploratory multicenter baseline survey to assess the baseline knowledge, attitudes and perceptions of oncology as a profession among final-year medical students in Kenya. The study was implemented across accredited Kenyan medical universities, where final-year medical students represent the immediate pipeline into internship and postgraduate training programs.

### Study population and sampling

Eligible participants were final-year Bachelor of Medicine and Bachelor of Surgery students in recognised Kenyan medical schools at the time of data collection. A census approach was used to maximise representativeness, with all final-year students invited to participate.

### Data collection tool

Data were collected using a structured questionnaire designed in Qualtrics. The collected information tool was adapted to local medical students’ specialty preferences and oncology-related knowledge, with contextual modification for the Kenyan setting. The questionnaire comprised four domains:

1. Sociodemographic characteristics (age, gender).
2. Career intentions (specialty preferences, postgraduate training plans, factors influencing career choice).
3. Oncology-specific knowledge and exposure (undergraduate oncology teaching, clinical rotations, knowledge of cancer burden, available oncology workforce).
4. Attitudes and perceptions toward oncology as a career (interest, perceived barriers, perceived role of oncology in Kenya’s health system).

The survey included both closed-ended questions (Likert scale, multiple-choice) and open-ended responses to capture nuanced perspectives.

### Data collection procedures

The survey link was distributed electronically via class representatives and student WhatsApp groups between 1st April and 30th May 2025. Participation was voluntary and anonymous. Two reminder messages were sent over a 3-week interval to enhance response rates.

### Data management and analysis

Responses were downloaded securely from Qualtrics into Microsoft Excel and analysed using SPSS version 27 (IBM Corp, Armonk, NY). Descriptive statistics were used to summarise participant characteristics and key study variables. Categorical variables were presented as frequencies and percentages. Bivariate analysis was performed to assess associations between independent variables (e.g., clinical exposure, curriculum rating, workshop attendance and awareness of training) and outcomes of interest, including knowledge, confidence and career interest in oncology. The chi-square test was used to evaluate associations between categorical variables. A *p*-value of <0.05 was considered statistically significant.

### Ethical considerations

Ethical approval was obtained from the Mount Kenya University Ethics Review Committee (Approval No. MKU/ISERC/5342). Permission was also sought from participating medical schools. Informed consent was embedded in the first page of the Qualtrics survey, and only consenting participants proceeded. No personal identifiers were collected.

## Results

### Participant demographics

**Figure 1.**
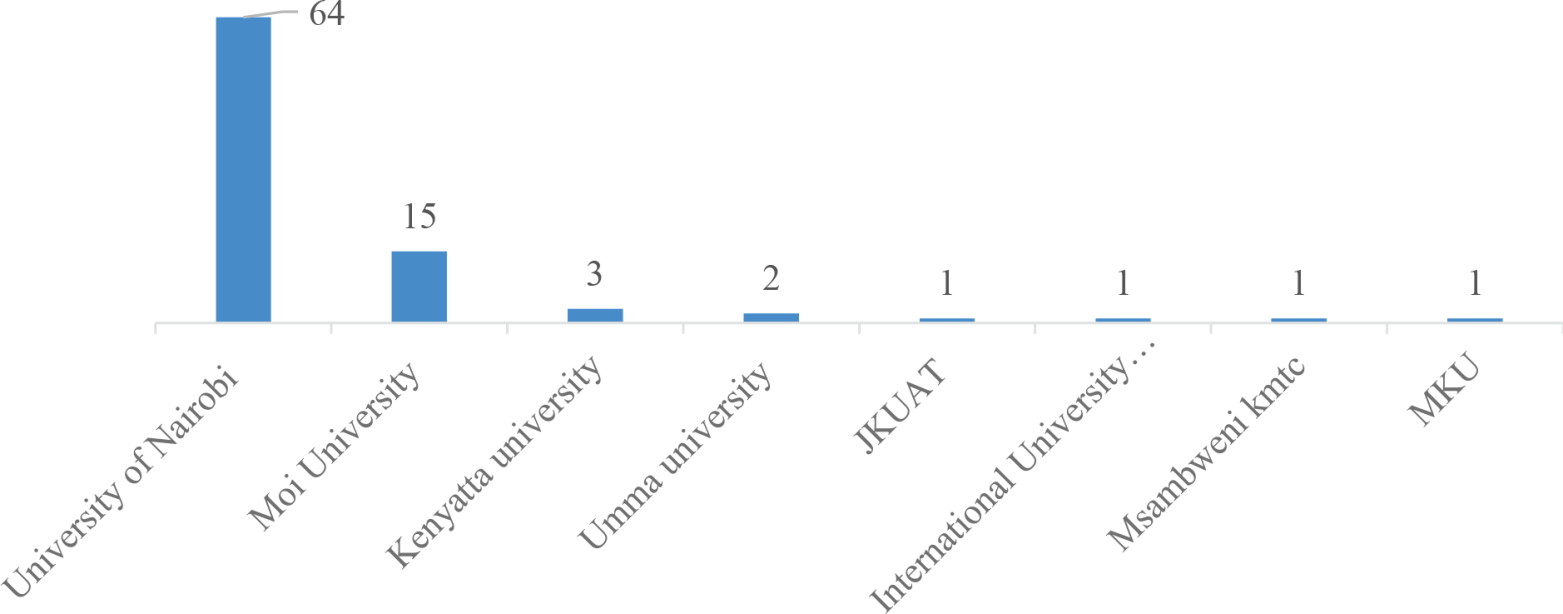
Number of respondents per institution.

A total of 89 responses were received from final-year medical students across various universities in Kenya, as well as one from outside the country. After excluding incomplete forms, 88 questionnaires were analysed. Respondents represented several institutions, with the majority from the University of Nairobi. Specifically, 73% (*n* = 64) were from the University of Nairobi, 17% (*n* = 15) from Moi University, 3% (*n* = 3) from Kenyatta University, 2% (*n* = 2) from Umma University and 1% each (*n* = 1) from Mount Kenya University, Jomo Kenyatta University of Agriculture and Technology, Msambweni KMTC and the International University of Africa–Khartoum as shown in Figure 1. Gender distribution was equal, with 50% (*n* = 44) female and 50% (*n* = 44) male. The mean age was 25 years across both groups.

### Students’ personal experiences with cancer

**Figure 2.**
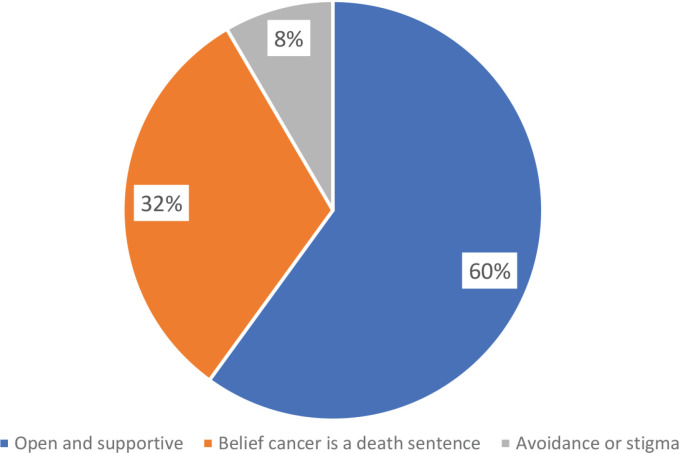
Perception of cancer diagnosis.

A majority of respondents reported that they had a close family member or friend diagnosed with cancer. Specifically, 77% (*n* = 68) indicated ‘Yes,’ while 23% (*n* = 20) reported no such experience. Students described varied family and community reactions to cancer diagnoses. The most common response was openness and support, reported by 60% (*n* = 57). About one-third, 32% (*n* = 30), perceived that cancer was viewed as a death sentence, while 8% (*n* = 8) experienced avoidance or stigma, as shown in Figure 2.

In terms of influence on perception of cancer on oncology as a career, personal or family experience with cancer shaped perceptions for many respondents. Sixty-one percent (*n* = 54) stated that their experience influenced how they viewed cancer or oncology as a career. Conversely, 37% (*n* = 32) indicated no influence, while 3% (*n* = 2) reported mixed feelings. Similarly, most students believed that cultural beliefs or taboos affected how their communities responded to cancer diagnoses. 81% (*n* = 71) answered ‘Yes,’ while 19% (*n* = 17) felt that cultural beliefs or taboos did not play a significant role.

### Oncology baseline knowledge and exposure among medical students

Most medical students had completed their oncology clinical rotation 73% (*n* = 64), but participation in oncology-focused workshops, seminars or continuing medical education was expectedly low. Only 30% (*n* = 26) of respondents had attended such oncology-focused workshops, while 70% (*n* = 62) had not.

When asked about their baseline knowledge of cancer incidence in Kenya, the response varied widely among students. Just over half, 53% (*n* = 47), reported that they were ‘not sure’ of the annual cancer incidence. Thirty-eight percent (*n* = 33) selected the correct range of the estimated 40,000–50,000 new cancer cases annually in Kenya, while 9% (*n* = 8) underestimated the figure at less than 10,000 cases. Also, when asked to identify common cancers in Kenya, prostate cancer was the most frequently identified, reported by 26% (*n* = 83) of responses. Esophageal cancer and Kaposi sarcoma were each cited by 23% (*n* = 65 and *n* = 74, respectively). Cervical cancer was identified by 20% (*n* = 25), while breast cancer was noted by 8% (*n* = 75).

Students also indicated their knowledge of treatment modalities available in Kenya, in which chemotherapy was the most frequently recognised option, selected by 29% (*n* = 73). Radiotherapy was closely identified at 28% (*n* = 72) while immunotherapy was mentioned less frequently, at 21% (*n* = 55). Interestingly, traditional medicine was also acknowledged by 8% (*n* = 20) of respondents as a form of cancer treatment in Kenya. Regarding their views on oncology training in medical school, the majority of the students (86%, *n* = 76) agreed that oncology training in undergraduate medical education should be strengthened.

Of note, most students 84% (*n* = 74) were aware that oncology is a recognised medical specialisation in Kenya, but awareness of the availability of local postgraduate oncology programs was mixed, with 42% (*n* = 37) aware of such training opportunities, whereas 33% (*n* = 29) were not and 25% (*n* = 22) reported being unsure. Furthermore, the assessments of oncology content in their medical school curriculum were varied, with the majority rating as ‘average,’ reported by 42% (*n* = 37). Thirty-six percent (*n* = 32) rated the content as ‘good,’ while 9% (*n* = 8) rated it ‘excellent.’ Eleven percent (*n* = 10) judged it ‘poor’ and 1% (*n* = 1) rated it ‘terrible.’

### Student perceptions of oncology as a career: motivators and barriers

When asked about the factors that shaped their perception of oncology as a future profession, clinical exposure to oncology and patient outcomes were the most frequently cited influences, each mentioned by 26% (*n* = 71). Strategies suggested by respondents to attract medical students to oncology as a career include increased clinical exposure 32% (*n* = 79), mentorship opportunities 32% (*n* = 77), expanding residency slots 23% (*n* = 55) and financial or career incentives 14% (*n* = 33).

**Figure 3.**
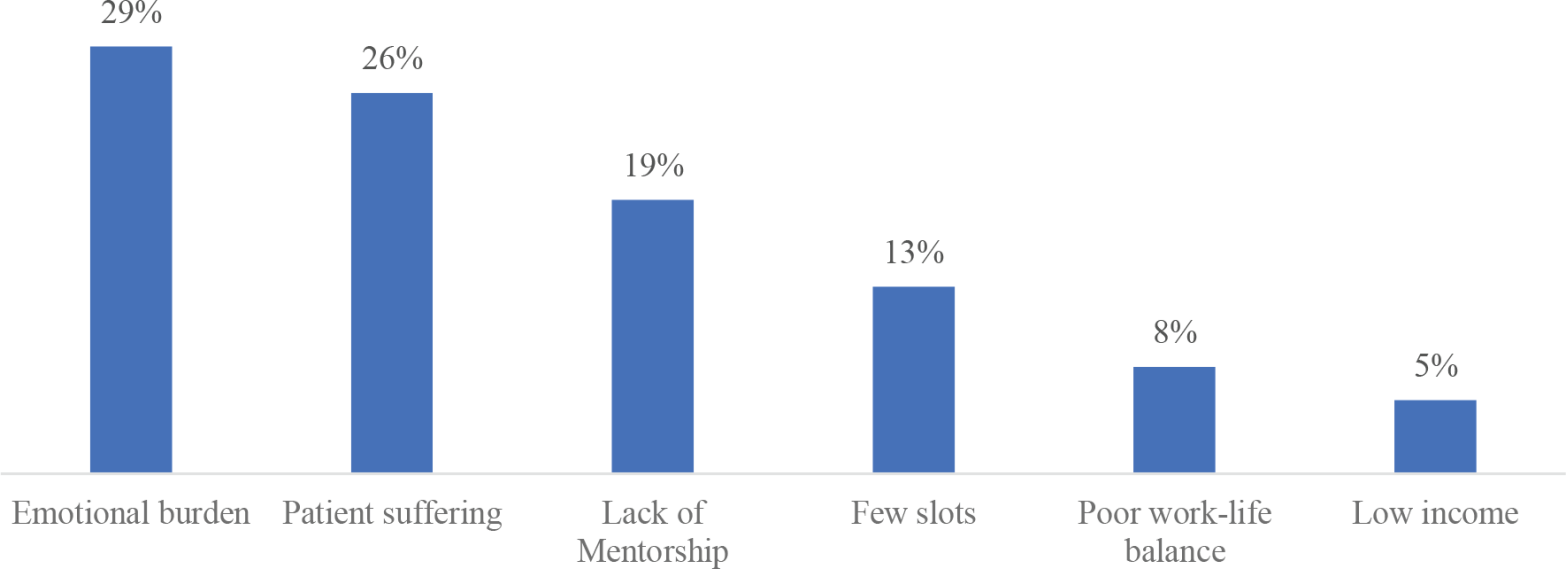
Factors discouraging pursuit of oncology as a career.

While factors discouraging the pursuit of oncology as a career among the students include emotional burden 25% (*n* = 64), patient suffering 22% (*n* = 57), lack of mentorship 16% (*n* = 41) and poor work–life balance 6% (*n* = 16), as shown in Figure 3. Consequently, when asked if they would consider specialising in oncology, 25% (*n* = 22) stated ‘yes,’ about half 51% (*n* = 45), responded ‘maybe’, while 24% (*n* = 21) indicated they would not consider a career in oncology

### Statistical analysis

The bivariate analysis highlights key drivers of oncology knowledge and confidence among Kenyan medical students, as well as gaps in factors influencing career interest. Three significant positive associations were observed. Completion of an oncology clinical rotation was associated with higher confidence in managing cancer patients (*p* = 0.013). Attendance at oncology-focused workshops was linked to improved knowledge of cancer incidence in Kenya (*p* = 0.042). The strongest association was between curriculum quality and confidence (*p* < 0.001), with students rating their oncology training more highly, reporting greater confidence in clinical care. In contrast, two key negative findings emerged. First, none of the examined factors, including clinical exposure and awareness of training opportunities, were significantly associated with interest in pursuing oncology as a career.

Second, knowledge of cancer incidence alone was not associated with increased confidence in managing patients (*p* = 0.341). Overall, while education and exposure improve competence, they do not necessarily translate into career interest in oncology. These findings are summarised in Table 1.

## Discussion

This study represents one of the first exploratory multicenter baseline study to explore the baseline knowledge, attitudes and perceptions of oncology among final-year medical students in Kenya. Our findings reveal that while the majority of students recognise the importance of oncology in Kenya’s health system, only a minority consider pursuing it as a career.

Knowledge of country-level cancer incidence and treatment modalities was mixed, with many students uncertain of the actual burden of disease; however, encouragingly, most had completed an oncology rotation and expressed that undergraduate oncology training should be strengthened. Nonetheless, barriers such as limited mentorship, emotional burden and poor awareness of postgraduate pathways continue to undermine oncology’s career attractiveness. These findings are consistent with studies from Uganda and elsewhere showing that medical students often gravitate toward core specialties such as internal medicine, surgery, paediatrics and obstetrics/gynaecology, while oncology remains underrepresented in career choices [13, 14]. Similar trends have been observed in Nigeria, where low exposure to oncology correlated with poor career uptake [10], and in Pakistan, where interest was likewise minimal [15]. Notably, modifiable factors such as mentorship exposure and awareness of training pathways were identified as key predictors, highlighting actionable opportunities for workforce development interventions.

By contrast, in high-income countries, although oncology is not a dominant specialty, modestly higher interest has been reported, often driven by structured rotations, mentorship and research opportunities [16]. Across sub-Saharan Africa, oncology remains critically understaffed, with many countries hosting only a handful of specialists and some none at all [6]. Kenyan surveys mirror these findings, showing low student preference for oncology despite the high national cancer burden [12].

Local training and fellowships in medical oncology, radiation oncology and gynaecological oncology and nursing oncology programs have been initiated at the local Kenyan universities, such as the University of Nairobi, Moi University, Aga Khan University, alongside the creation of regional cancer centers, such as the Garissa regional cancer center, to address these gaps in improving postgraduate training in oncology, as well as nursing oncology [17–19]. Our study adds nuance by providing student perspectives, highlighting modifiable barriers such as insufficient mentorship, unclear postgraduate pathways and limited curriculum integration, factors that could be directly targeted to stimulate interest in oncology.

The implications for workforce development in Kenya are significant. The National Cancer Control Strategy (2023–2027) acknowledges workforce strengthening as a priority, yet the reality remains stark: fewer than 100 oncologists serve a population of over 50 million [8, 20]. However, unless medical students perceive oncology as a viable and supported career pathway, these initiatives may struggle to meet enrollment targets. Our findings suggest that integrating oncology more systematically into undergraduate curricula, expanding rotations at cancer centers, fostering mentorship through student interest groups and providing clearer postgraduate pathways could be high-impact, low-cost strategies to attract future oncologists. Highlighting diverse career tracks, including medical oncology, radiation oncology, surgical oncology, palliative care and research, may also broaden student interest and encourage uptake.

The strengths of our study include its multi-university representation, offering the first baseline insights into Kenyan medical students’ oncology career perceptions. The use of an electronic survey platform enhanced reach and convenience across diverse institutions. However, limitations include reliance on self-reported data, which may introduce recall and social desirability bias and the cross-sectional design, which limits causal inference. Additionally, the unequal distribution of responses across institutions, with dominance from the University of Nairobi, may influence generalisability.

This study has several limitations. First, the sample size was modest and may limit statistical power. Second, there was disproportionate representation from a single institution, which may introduce institutional bias and limit generalisability. Third, the cross-sectional design precludes causal inference.

However, this study provides important exploratory insights into oncology career perceptions among Kenyan medical students and serves as a foundation for larger, nationally representative studies.

Looking forward, future studies should longitudinally track Kenyan medical graduates to evaluate whether early perceptions translate into actual uptake of oncology as a career. Comparative research with neighbouring East African countries would further contextualise regional workforce trends. Importantly, intervention studies that test oncology modules, structured rotations or mentorship programs are needed to determine their effectiveness in improving student interest and eventual career choice.

## Conclusion

Interest in oncology as a career preference remains underrepresented among Kenyan final-year medical students, despite widespread recognition of its importance in tackling the cancer burden of Kenya. Knowledge gaps, inadequate exposure and the absence of mentorship contribute significantly to this trend. Strengthening undergraduate oncology training, creating visible career pathways and fostering supportive mentorship structures will be essential in cultivating the next generation of oncologists aligned with Kenya’s cancer control priorities.

## Conflicts of interest

None to declare.

## Funding

None to declare.

## Figures and Tables

**Table 1.** Bivariate analysis of factors associated with oncology knowledge, confidence and career interest.

Independent variable	Outcome variable	Statistical test	p-value	Interpretation
Clinical rotation	Confidence in managing cancer patients	Chi-square	0.013	Significant – higher confidence with rotation
Workshop attendance	Knowledge of cancer incidence	Chi-square	0.042	Significant – improved knowledge
Curriculum rating	Confidence in managing cancer patients	Chi-square	<0.001	Strong significant – better curriculum linked to higher confidence
Awareness of training	Recognition of oncology specialty	Chi-square	0.015	Significant – improved recognition
Awareness of training	Career interest	Chi-square	0.357	Not significant
Cancer experience	Career interest	Chi-square	0.769	Not significant
Clinical rotation	Career interest	Chi-square	0.407	Not significant
Workshop attendance	Career interest	Chi-square	0.106	Not significant
Knowledge of incidence	Confidence in managing cancer patients	Chi-square	0.341	Not significant
